# Video Monitoring Application of CMOS 4T-PPD-APS Under γ-ray Radiation

**DOI:** 10.3390/s19020359

**Published:** 2019-01-17

**Authors:** Shoulong Xu, Shuliang Zou, Yongchao Han, Yantao Qu, Taoyi Zhang

**Affiliations:** 1 School of Resource Environment and Safety Engineering, University of South China, Hengyang 421001, China; xusl@usc.edu.cn; 2 Department of Engineering Physics, Tsinghua University, Beijing 100084, China; 3 China Institute of Atomic Energy, Beijing 102413, China; hanyongchao@ciae.ac.cn (Y.H.); quyantao@ciae.ac.cn (Y.Q.); 4 Beijing Institute of Control Engineering, Beijing 100084, China; zhangtaoyi@sunwiserobot.com

**Keywords:** ionizing radiation, radiation response, radiation interference, 4T-PPD-APS

## Abstract

In this paper, we discuss the potential use of four transistor active pixel sensor (4T-APS) as a video monitor at a nuclear accident site with a high level of γ radiation. The resistance and radiation responses to γ radiation were investigated by radiation experiments using ^137^Cs and ^60^Co γ-ray sources. The radiation resistance of 4T-APS was studied by testing the mean and the maximum dark current of the sensors after irradiation. A random spatial distribution of radiation response events was observed upon analyzing these events on the video images in a given time during irradiation. The background dependence of the 4T-APS was also studied by comparing the grayscale incremental value of the images with different color and grayscale backgrounds: the radiation response events were obvious on the images with a background having a smaller grayscale value or a deeper color. Finally, the color saturation and resolution of the images were tested using a vector oscilloscope and a test card. When the total ionizing dose was less than or equal to the damage threshold, no significant performance deterioration of 4T-APS was observed in an environment with sufficient light.

## 1. Introduction

In a nuclear facility accident, radiation could be detrimental to the applied diagnostic equipment, such as video image systems as well as electronic components of the detection systems. In particular, γ-rays are considered in the radiation environment of nuclear facility accidents because their level of energy is high and their potential for damage to tissue is extremely high [1,2]. Previous research has revealed that the maximum dose rate in the Fukushima accident reached 650 Sv/h. A complementary metal oxide semiconductor (CMOS) active pixel sensor (APS) video monitor is relatively cheap, consumes little power, has strong environmental adaptability, has strong radiation resistance, and is sensitive to radiation; therefore, it has been applied to radiation environment monitoring [3,4,5,6].

Compared with the charge-coupled device pixel sensor, CMOS APS has a considerable advantage in radiation resistance [7], but the defects degrade the performance of APS because of the dark current signal; thus, the appearance of individual pixels with a high dark current is affected by the total ionizing dose (TID) effect, dose rate, and exposure time [8]. Cohen and David found that ^60^Co γ photons have a serious influence on the dark current of CMOS APS [9]; Goiffon carried out a series of experimental studies and theoretical analyses on the radiation TID effect and single-event effect of CMOS APS [10,11,12,13,14,15,16,17,18]. The appearance of the interference of individual pixels resulted in a high value (“hot pixels”), which was clearly generated by the high-dose-rate γ radiation [19,20]. To expound the radiation effect on APS monitoring in radiation exposure, we conducted an experimental study using γ radiation [21,22]. In this letter, we present the first result on the radiation resistance of CMOS APS with four transistors (4T) and a pinned photodiode (PPD) obtained by an analysis of radiation damage experiments. We studied the radiation resistance of APSs to γ irradiation by testing their operation life and dark current, which is closely related to TID. Moreover, the sensor performance was studied by comparing the radiation response of images with different backgrounds and testing the chromaticity and the image resolution at different γ-ray irradiation dose rates.

The rest of this paper is organized as follows. The experimental setup and the data processing methods are detailed in Section 2. The experimental result and the data processing are described in Section 3. Our conclusions are presented in Section 4.

## 2. Experiments

### 2.1. Initial Parameters of Image Sensors

Commercial sensors with four transistors and a pinned photodiode were used in the experiments. We used diagonal 6.4-mm (Type 1/2.8) CMOS active pixel-type image sensors with a square pixel array and approximately 2.43 M effective pixels. The manufacturer of sensors is SONY, and the model is IMX 222LQJ-C (more details could be obtained from product datasheet, Sony Corporation, Japan, Tokyo). The bit depth of the sensors was 8. The chips operated with analog 2.7 V, digital 1.2 V, and interface 1.8 V triple power supplies. The R, G, and B primary color pigment mosaic filters were adopted in the sensors. High radiation resistance and low dark current and no smear could be achieved [4,21]. The integration time of all of the sensors was set to 1/25 s. The video was recorded at a frame rate of 25 fps in a video format with a HikVision (Zhejiang, China) model DS-7804N-K1/C network work recorder (NVR) with four signal acquisition channels during the γ-ray irradiation. The power supply voltage was DC 12 V. Video files were imported using MATLABR 2014a (Math Works Inc., Natick, MA, USA) and then split into individual frames.

### 2.2. ^137^Cs Source Experimental Setup

**Figure 1:**
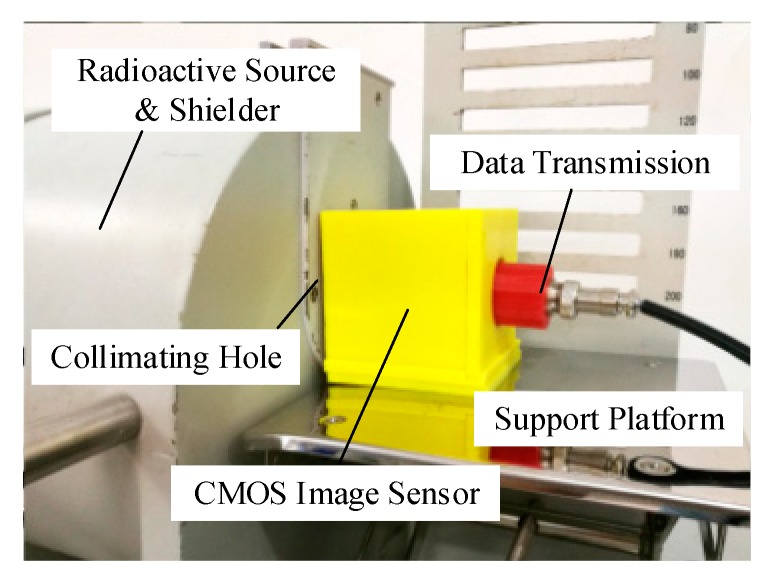
^137^Cs γ-ray source radiation experimental device.

A ^137^Cs γ-ray source was used in the experiments of the radiation response with the energy of 0.662 MeV. The shielder and collimating hole setup was the auxiliary structure in the experiments, which performed functions such as radiation protection and generated collimation photon rays. The diameter of the collimating hole was 1 cm, and the photon fluence was 657 photons/cm·s^2^. Sensor samples were placed in the holder positions. The maximum background value (contribute by noise) of the pixels was 3 (gray) without interference from visible light, infrared rays, and ultraviolet rays. The experimental temperature was 21°. The experimental setup is shown in Figure 1.

### 2.3. ^60^Co Source Experimental Setup

**Figure 2:**
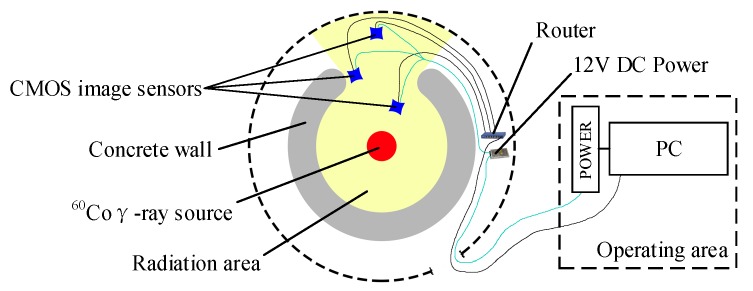
Schematic representation of ^60^Co γ-ray experimental system.

A columnar ^60^Co γ-ray source was used in the experiments with photon energies of 1.17 and 1.33 MeV. The activity of the radioactive source was 90 kCi. The γ-ray dose rate as a function of time was measured with a silver dichromate chemical dosimeter. The dose rate is determined by the positions of the resulting experimental points. The positions closer to the source represent higher dose rates. The peripheral circuits are shielded by a tungsten shield, and the sensor is irradiated through an aperture in the shield with a diameter greater than that of the sensor. Besides of the sensors, all the other experimental setups are located in the non-radiation region. One group of the total ionizing radiation resistance experiments was conducted at 154.5 Gy/h. The other group of radiation response experiments was conducted at four different dose rates, namely 38 Gy/h, 78 Gy/h, 234 Gy/h, and 557 Gy/h. All of the experimental samples were placed in an experimental box with a video test card that could insulate samples from visible light, infrared rays, and ultraviolet rays. The color temperature in the box was 6000 K, and the mean illumination was 3744 lux. Figure 2 shows a schematic representation of the experimental system. 

### 2.4. Data Processing Methods

The recorded video data were processed on a PC by using video editing software, which was used to select the videos recorded during and after the irradiation and to transform the videos into images (25 fps according to the sampling rate; each frame was an image). All of the images were transformed into an 8-bit gray value matrix for the analysis of the image grayscale values; therefore, we obtained an output signal called grayscale value from 0 to 255; the unit was “gray.” The radiation effect of γ-rays on the video data was evaluated by calculating the increase in the gray value before and after the radiation.

The grayscale incremental value overlapping matrix (*D_k(m×n)_*) at the radiation dose rate of k was calculated as follows:

(1) $$D_{k\left(m\times n\right)}=\sum_{l=1}^{h}\left(E_{l\left(m\times n\right)}-I_{\left(m\times n\right)}\right),$$

where *I_(m×n)_* is the grayscale value matrix of the image before irradiation, *E_l(m×n)_* is the grayscale value matrix of the *l*-th image at the radiation dose rate of *k*, and *h* is the number of frames.

The mean grayscale incremental value (*S_k_*) of the selected images at the radiation dose rate of *k* was calculated as follows:

(2) $$S_{k}=\frac{1}{MN}\sum_{j=1}^{j=M}\sum_{i=1}^{i=N}\left(E_{i,j}-I_{i,j}\right),$$

where *I_i,j_* is the grayscale value of the *i*-th pixel in the *j*-th frame before irradiation, *E_i,j_* is the grayscale value of the *i*-th pixel in the *j*-th frame at the radiation dose rate of *k*, *M* is the number of frames, and *N* is the pixel count.

The dark current was calculated with the help of functions from EMVA Standard 1288; the functions could be shown as follows:

(3) $$I_{dark}=\frac{\frac{1}{2N}\sum_{i=1}^{N}\left(E_{i,j}+E_{i,j+1}\right)-BL}{\tau_{in}\cdot \sum_{i=1}^{N}\left(E_{i,j}+E_{i,j+1}\right)/\sum_{i=1}^{N}{\left(E_{i,j}-E_{i,j+1}\right)}^{2}},$$

where *BL* is the black level, and *τ_in_* is the integration time (1/25 s for all of the sensors).

## 3. Discussion of Data Processing and Results

### 3.1. Radiation Resistance

The analysis of the radiation resistance of 4T-APS was based on the ^60^Co γ-ray source experiment; all of the samples were irradiated during operation, and we used the same detector holder positions as those used during the irradiations. The dose rate at these positions was 154.5 Gy/h. The entire system was kept there for 2.5 h. All the sensor chips were dark folded to cut off visible light; thus, the frames were dark current frames. Each frame series was saved in the JPEG file format and contained 4000 frames.

**Figure 3:**
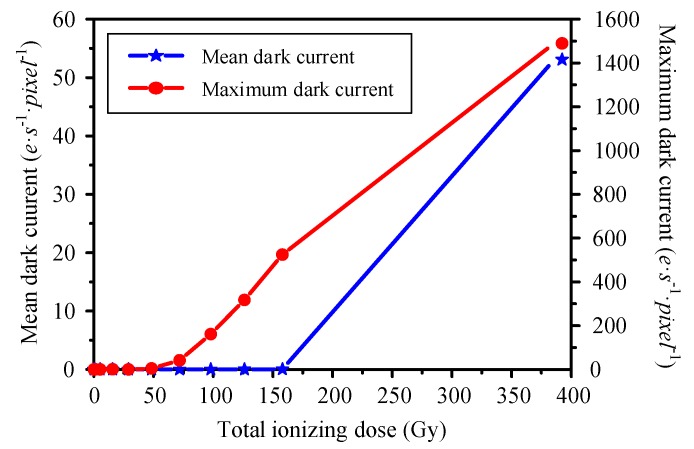
Histogram of mean and maximum dark current of images after different TID γ-ray irradiations.

The overall behavior of the mean dark current and the maximum dark current during irradiation is shown in Figure 3. We described the mean dark current and the maximum dark current as a function of the TID; there was a damage threshold in the TID effect. As shown in Figure 3, no obvious dark current change was exhibited before 50 Gy; the maximum dark current of the pixels began to increase after 50 Gy, but the mean value increased less; the dark current of several pixels began to increase sharply with a relatively large TID of more than 150 Gy. Therefore, all of the sensor samples had to be irradiated at less than 50 Gy during the radiation response experiments.

### 3.2. Radiation Response

**Figure 4:**
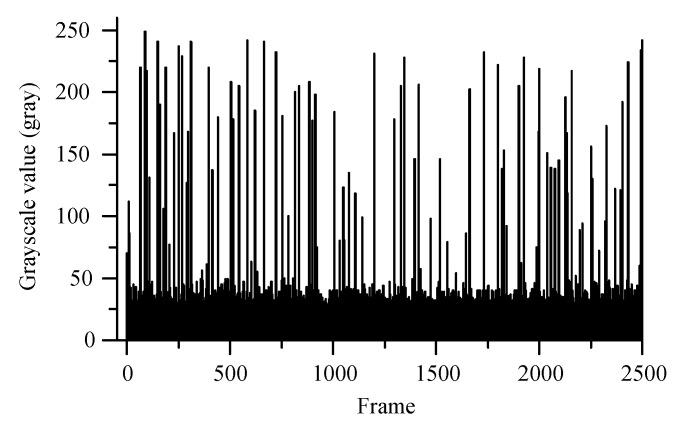
Histogram of maximum grayscale value of images during irradiation at a given time of 2500 s.

The radiation response analysis of 4T-APS was based on the results of the ^137^Cs γ-ray source experiment, the TIDs of all of the sensors were less than 1 mGy. The sensor samples were in operation throughout the experiment. The length of the selected video was 2500 s. Videos were transformed into frames (25 fps; each frame was saved in a JPEG file format image). Figure 4 shows the histogram of the maximum grayscale value of 35,729 frames in 2500 s. A grayscale value larger than the background indicated that radiation response events appeared on each image. The maximum value of each image was greater than 20 (gray) during the ^137^Cs γ-ray radiation, and the peak value was a random occurrence from 20 to 249 (gray).

**Figure 5:**
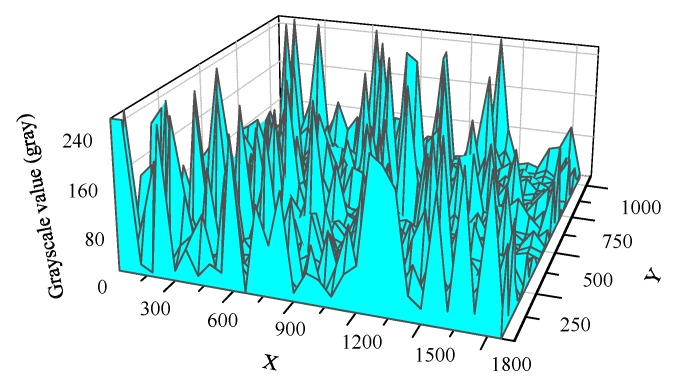
Mesh of overlapped radiation response events at a given time of 2500 s.

To study the spatial distribution of the response events, we overlapped the grayscale value of 35,729 frames from the 2500-s video. The mesh of the overlapped radiation response events is shown in Figure 5. It indicated that the radiation response events occurred throughout the pixel array region randomly and that many peaks were distributed randomly over the mesh. 

Figure 4 and Figure 5 show that the response pixels were randomly distributed in time and space. The grayscale value of the pixels in a region increased sharply when the radiation response events overlapped at the same time and the peak value was close to saturation. According to the study result of early research [20], the random distribution response events were generated by the γ-ray ionizing radiation. This radiation response characteristic enabled the CMOS APS to be disturbed by the γ-ray irradiation and had the potential to serve as a nuclear radiation detection and radiation warning device.

**Figure 6:**
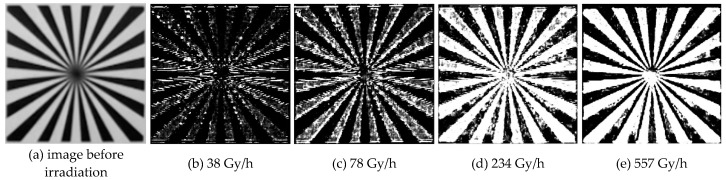
Comparison of radiation responses in black and white background regions at irradiation dose rates of 0, 38, 78, 234, and 557 Gy/h. Upon the overlapping of the grayscale value of 2000 frames, an obvious difference in the radiation response between the black and the white background regions was observed; the boundary was more obvious at a larger irradiation dose rate. The radiation response events generated in the white regions required irradiation at a higher dose rate.

The background dependence of the 4T APS was studied by using the video test card and the ^60^Co γ-ray source. The TIDs of all of the sensors were less than 30 Gy, and all of the sensors were in operation throughout the experiment. Figure 6a shows the video test image with black and white stripes before irradiation; the background grayscale value of the black regions was 25 (gray), and the value of the white regions was 200. With the help of function (1), the grayscale values of 2000 frames for the irradiation with each different dose rate were overlapped and drawn as grayscale maps, as shown in Figure 6b–e. The radiation response events were mainly generated in the black region. In the case of irradiation at a relatively large dose rate, the number of events was increased and the black and white boundaries of the response regions were more obvious. Meanwhile, the radiation response events only appeared in the white region when the radiation dose rate was greater than 234 Gy/h.

**Figure 7:**
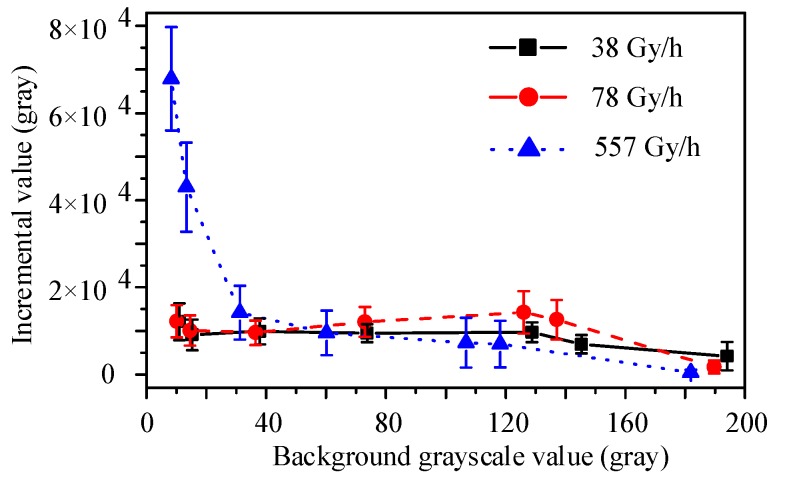
Mean grayscale incremental value of 4T-APS with different grayscale backgrounds at the irradiation dose rates of 38, 78, and 557 Gy/h.

**Figure 8:**
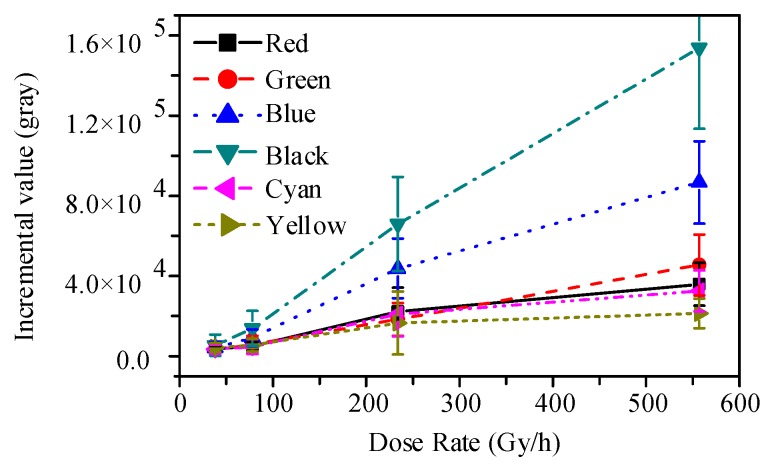
Mean grayscale incremental value of 4T-APS with different color backgrounds at the irradiation dose rates of 8, 78, and 557 Gy/h.

The mean grayscale incremental values obtained for the 5-min video of γ-ray irradiation were calculated using function (2). The relationship between the grayscale incremental value and the irradiation dose rate for different colors and different grayscale backgrounds is shown in Figure 7 and Figure 8. 

The curves shown in Figure 7 indicate that images with a smaller grayscale background have a greater incremental value. At the irradiation dose rates of 38 and 78 Gy/h, the incremental value of the images with a background grayscale value of less than 129 (gray) was greater than the value for frames with a larger background grayscale. The incremental value of images with a background grayscale value of less than 38 had a distinct difference at the irradiation dose rate of 557 Gy/h. This was attributed to the fact that the radiation response in the images could be described as an additional value of the background. Pixels with a lower background grayscale had more space to store and transfer the charges generated by the γ-ray ionizing irradiation, but the grayscale value of pixels with a higher background could easily reach saturation (255 gray).

The curves shown in Figure 8 indicate that images with different color backgrounds had different radiation responses and the difference became more obvious when the irradiation dose rate was increased. The incremental value of the blue and the black background images increased sharply with a larger dose rate, but a similar increase amplification was not seen for the images of other color backgrounds. This was attributed to the fact that the intensity of visible light reflected by each type of color background was different at the same illumination. This implied that the space-charge region of the blue and black background pixels had more space than the other color background pixels to store and to transfer the radiation-induced charges. Thus, we concluded that there was a weak radiation response in light color background images but a serious effect in the deep ones.

**Figure 9:**
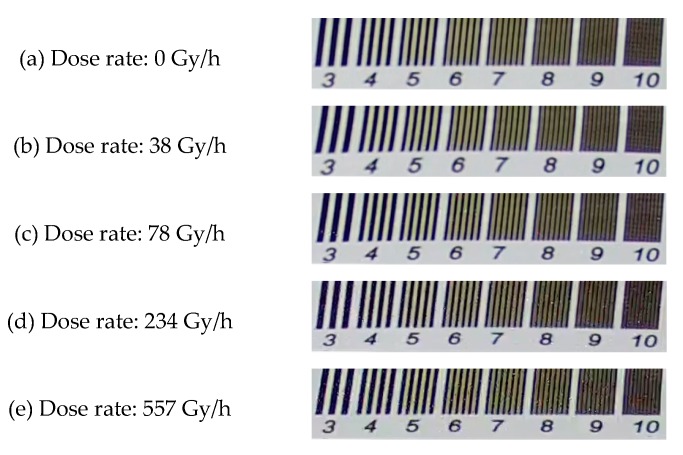
Resolution test images of 4T-APS at irradiation dose rates of 38, 78, 234, and 557 Gy/h. Noises appeared in these images; no blur and resolution decrease was observed in these images, and the boundary of the black and white stripes became more obvious during the γ-ray irradiation.

White noise appeared in the resolution test, but there was no blur and resolution decrease in the images, and the boundary of the black and white stripes became more obvious at the start of the irradiation, as shown in Figure 9. Thus, we concluded that, under sufficient light, there was no significant performance deterioration of 4T-APS before the TID reached the damage threshold.

## 4. Conclusions

In this work, we investigated the radiation resistance and the radiation response of CMOS 4T-PPD-APS. We discussed the potential use of 4T-APS as a video monitor at a nuclear accident site with a high level of γ radiation. The radiation-induced response events were spatially and randomly distributed on the images captured during irradiation. The radiation response characteristic enabled the CMOS APS to be disturbed by the γ radiation. The radiation response events were mainly generated on the images with a smaller grayscale background or a deep color background such as black and blue. The color saturation of the images was improved slightly with the γ radiation, but a more obvious change occurred under irradiation at a high dose rate. While white noise appeared in the resolution test images, no blurring and resolution decreases occurred on the images during the irradiation.
